# Cardiopulmonary Bypass, Inflammation and How to Defy it: Focus on Pharmacological Interventions

**Published:** 2012

**Authors:** Ali Dabbagh, Samira Rajaei, Ayad Bahadori Monfared, Ali Asghar Keramatinia, Korosh Omidi

**Affiliations:** a *Anesthesiology Research Center, Shahid Beheshti University of Medical Sciences, Tehran, Iran.*; b *Deptartment School of Medicine, Tehran University of Medical Sciences, Tehran, Iran*; c *Candidate, Epidemiology Department. School of Public Health, Shahid Beheshti University of Medicine, *; d *Tehran, Iran.*; e *Shahid Beheshti University of Medicine, Tehran, Iran.*

**Keywords:** Cardiopulmonary, Inflammation, Pharmacological, Technical strategies, Anti-inflammatory method

## Abstract

One of the most common health problems are diseases of the cardiovascular system with a great bulk of disease burden; while a considerable number of cardiac patients undergo cardiac surgery; cardiac surgical procedures with cardiopulmonary bypass (CPB) are nowadays among the top list of surgical procedures.

More than half of a century has passed since the introduction of total cardiopulmonary bypass (CPB). One of the main untoward effects of CPB is systemic inflammation; causing an “acute phase reaction” responsible for the production of other unwanted postoperative complications.

The humoral and cellular components of the immune system are among the main parts of these compensatory mechanisms. There are a number of therapeutic agents used to suppress this inflammatory process.

Since CPB is composed of a multitude of items, there are many studies assessing the possible methods and therapeutics for prevention or treatment of inflammation in patients undergoing CPB.

According to a conventional classification, the anti-inflammatory methods are classified as either pharmacologic strategies or technical strategies. The pharmacologic strategies are those with the usage of one or more therapeutic agents; while the technical strategies are those that try to modify the CPB techniques. However, in this manuscript, the main pharmacological strategies are discussed.

## Introduction

One of the most common health problems are diseases of the cardiovascular system with a great bulk of disease burden; while a considerable number of cardiac patients undergo cardiac surgery; in such a way that cardiac surgical procedures with cardiopulmonary bypass (CPB) are nowadays among the top of the list of surgical procedures (1).

More than half of a century has passed since the introduction of total cardiopulmonary bypass (CPB) for the first time for cardiac surgery and “is now used successfully thousands of times each day worldwide”. One of the main untoward effects of CPB is systemic inflammation. So, among the unwanted side effects of CPB, systemic inflammation could be mentioned as one of their greatest ones; causing an “acute phase reaction” responsible for the production of other unwanted postoperative complications (2). 

**Table 1:** The total number of papers reviewed and their characteristics

	**Topic**	Number of orticles*	**Evidence level**	**Year of publication**
1	General issues	1-13	A-B	1997-2011
2	Aprotinin	14-37	A-C	1966-2010
3	Corticosteroids	35-44	A-B	1995-2011
4	Antioxidants	45-52	A-C	2001-2010
5	Phosphodiesterase Inhibitors	53-60	B-C	1999-2010
6	The complement cascade	61-65	A-B	1998-2010
7	Miscellaneous Agents	66-86	A-B	1997-2012
8	Non-pharmacologic strategies	87-111	A-C	1994-2010

* The number of the articles related to this topic

 The humoral and cellular components of the immune system are among the main parts of these compensatory mechanisms and at the same time, there are a number of therapeutic agents used to suppress this inflammatory process. Hence, we know that a series of interactions occurs during CPB, finally leading to the “activation of leukocytes and endothelial cells” (2-4). 

The CPB is composed of a multitude of items; so, due to the great number of its different parts and also, due to the very great number of resulting interactions between these varying components, there are many studies assessing the possible methods for prevention of inflammation in patients undergoing CPB; In addition, new therapeutic modalities may emerge after studies are performed regarding these many different variables.

 According to a conventional classification, the anti-inflammatory methods are classified as either pharmacologic strategies or technical strategies. The pharmacologic strategies are those engaged with the usage of one or more therapeutic agents; while the technical strategies are those that try to modify the CPB techniques. 

*Objectives*

One of the main untoward effects of CPB is systemic inflammation with its cellular and humoral components (5-6).

The CPB is composed of a multitude of elements; so, due to the presence of these elements and their interactions, a wide range of new therapeutic modalities could be used during the CPB procedure as potential locations for defying inflammation (7-10). However, these interventions are classified in one of the two main categories: pharmacologic strategies and technical strategies (11-13).

We studied the current evidence regarding pharmacological strategies used to defy inflammatory response in CPB. Hence, this summarizing review aims to consider different pharmacologic methods used for the treatment of inflammation in patients undergoing CPB for cardiac surgeries.

*Methods of the review*

For the review process, the Medline search engine was used which was covering articles from 1995 up to now through the following link: http://www.ncbi.nlm.nih.gov/pubmed/.

In Medline, the keywords were searched using Medical Subject Headings (MeSH) which at first, the following subheadings were selected, which resulted in 1010 citations at the time of the study:

*“Cardiopulmonary Bypass” and “inflammation”*

Then, they were searched separated by AND each time. Then, in each search, the search was completed with the other keywords, for example including “aprotinin”, “glucocorticoids”, “antioxidants”, “Phosphodiesterase Inhibitors” and “Complement System Proteins”.

In the remaining section of the manuscript, the main pharmacological strategies are discussed.

*Aprotinin*

The Medline search at the time of the study resulted in 77 citations for these phrases:

*“Cardiopulmonary Bypass” AND “inflammation” AND “aprotinin”*

Aprotinin is a bovine version of the small protein basic pancreatic trypsin inhibitor (known as BPTI), and has a high molecular weight, It is a very potent and nonspecific serine protease inhibitor, which inhibits the important fibrinolytic enzyme trypsin, as well as, its related important proteolytic enzymes. The trade name of aprotitn called “Trasylol”. For many years, aprotinin was administered as an anti-fibrinlytic agent, that decreased the amount of surgical bleeding. In European countries, in the final years of 1950s, aprotinin was used in order to attenuate hyperfibrinolytic conditions; although it had been discovered in 1936 (14).

This compound can inhibit a number of proteases, including trypsin, kallikrein and plasmin. In the 1960s, for the first time this compound agent was used as a therapeutic agent for the treatment of patients with acute pancreatitis. This was due to its effects in suppressing the proteases (14-17). There are a number of studies, especially during the last decade, demonstrating the role of lower aprotinin doses in decreasing the severity of post CPB systemic anti-inflammatory response syndrome (SIRS). This is probably due to the anti-inflammatory effects of the drug, which resemble the effects of corticosteroids. Also, there are some studies that demonstrate the anti-inflammatory effects of the drug on the release of “tumor necrosis factor-alpha” and “neutrophil integrin CD11b upregulation” (17-19). Besides, aprotinin can inhibit a few inflammatory agents including trypsin, chymotrypsin, plasmin and kallikrein. The related studies have mentioned a range of concentrations from about 125,000 IU/mL to 300,000 IU/mL for this anti-inflammatory action. These substances (*i.e. *trypsin, chymotrypsin, plasmin and kallikrein) are among the main compounds playing pivotal roles in the inflammation cascade and some are part of the contact system. 

Never the less, since 1980s, aprotinin was used experimentally and then clinically to reduce the CPB induced SIRS. In the first series of the cardiac operations, its use demonstrated significantly decreased postoperative bleeding and postoperative transfusion requirements (20- 22).

But, years later, in 2006, the studies published by Karkouti and colleagues (23-25) and also, by Mangano and colleagues (26-28), heavily questioned the safety of the drug worldwide. Those who favored aprotinin claimed that aprotinin treatment could significantly reduce the amount of blood transfusion and put a question mark on the methodology of the two latter studies. However, the critiques claimed that it could not benefit the patients regarding their clinical outcome in reducing morbidity or mortality. It could in best circumstances only be compared with the control groups; while, at the same time, blood transfusion is associated with increased morbidity and mortality (29).

Finally, a high quality study was designed in order to compare the results of aprotinin with the other tow analogs of lysine, namely tranexamic acid and aminocaproic acid. The study was performed in high risk patients who had undergone cardiac surgery. It was entitled the Canadian BART trial (Blood Conservation using Antifibrinolytics: a Randomized Trial in high-risk cardiac surgery patients). In November 2007, after about 2400 patients among the primary total population size of the study (*i.e*. 2900 patients) entered the study, the Data Safety Monitoring Board of BART trial decided to stop the study before the planned and determined time (30-32), due to the outcome of the patients till the time. Finally, the results demonstrated that aprotinin, when compared with the other tow antifibrinolytic agents (namely tranexamic acid and aminocaproic acid) was the most effective agent regarding its hemostatic effects; Furthermore, it could reduce the patient risk for severe postoperative hemorrhage and the need for postoperative use of blood and its products. Meanwhile the BART trial demonstrated that in patients who had received aprotinin, the one month risk of mortality was increased more than 50% , compared with the other patients (14, 32). After the primary results of the BART trial were gradually revealed, the US Food and Drug Administration (FDA) announced on November 5, 2007, that Trasylol (aprotinin) manufacturer (i.e. Bayer) should halt the marketing of the drug until more comprehensive studies would lead into a decision. This announcement has not been changed yet; though, there are newly emerged studies announcing controversial reports (14, 32-35). So, it seems that the story related to aprotinin uses and hazards will continue.

At the same time, we have to consider that according to the newest available meta-analyses, the use of aprotinin has no major effect on the proteins of acute phase of inflammation or on the cytokines related to inflammation in adult patients undergoing cardiac surgery with CPB.There fore, the present available evidence cannot support the use of aprotinin as anti-inflammatory agent per se (32, 36); though a number of studies still claim its anti-inflammatory effects (37).

*Corticosteroids*

The Medline search at the time of the study resulted in 39 citations for these phrases:

*“Cardiopulmonary Bypass” AND “inflammation” AND “glucocorticoids”*

When talking about inflammation, glucocorticoids are among the first drugs that come to the mind. This is however, the same fact for cardiac surgery. These agents have been used for more than 4 decades; while their related studies are not decisive yet.

The basis for using these agents in cardiac surgeries with CPB was the similarity in the clinical condition of the patients undergoing CPB with the patients experiencing infectious SIRS. However, the clinical usefulness of glucocorticoids in CPB is not as definitive yet, though many different clinical benefits of these drug category has been claimed for CPB (1, 35-41) and some have mentioned their possible harms (42-43). Even, some have claimed these drugs to be of no benefit for patients undergoing cardiac surgery with CPB (42-44).

As a general recommendation, the expert guidelines on CABG by the American Heart Association and the American College of Cardiology declared that glucocorticods are inexpensive and may decrease the hazards related to CPB related SIRS (38-41). Although the studies related to the benefits of glucocorticoids in CPB do not have definitive results, these agents are used in the daily practice of cardiac surgery in many centers worldwide (39-44).

*Antioxidants*

The Medline search at the time of the study resulted in 18 citations for these phrases:

*“Cardiopulmonary Bypass” AND “inflammation” AND “antioxidant”*

The process of myocardial ischemia and reperfusion, causes myocardial cell death, mainly through cellular apoptosis. Ischemia/reperfusion injury is the result of tissue ischemia beyond the upper tolerable cellular limits. As a rule, the cells tolerate a limited and defined ischemia period. Ischemic events exceeding this defined period, would incur ischemic insults (5). Ischemic times more than the tolerable period would result in a phenomenon named “ischemia/reperfusion injury” (6). The production of reactive oxygen species (ROS; also known as oxygen-free radicals) is the hallmark of ischemia/reperfusion injury (7, 45). Usually, the over-production of the ROS (*i.e. *ischemia reperfusion injury) happens after restoration of blood flow to the ischemic organ cells (46-47).

In one study, during the early few hours after CABG with CPB, the levels of “neutrophil gelatinase associated lipocalin” in plasma of patients was demonstrated to be an indicator of acute renal injury (48). Also, in another study, it was demonstrated that the release of endothelial nitric oxide and also, the plasma levels of nitrite oxide is strongly dependent on the method used for CPB (49).

In another study, an index called OXY-SCORE was presented. This score is an index which is a collective and brief demonstrator of the components of oxidative stress and its total status. The study demonstrated that this index has a good ability to predict the oxidative status of the patients (50). 

Among the many pharmaceuticals mentioned as antioxidants in patients undergoing CPB, Vitamins E and C and also, mannitol, allopurinol, and *N*-acetyl cysteine (*i.e. *the endogenous oxygen radical scavengers) are cited more than all the others (49-52). However, most human studies have failed to prove the benefit of routine administration of antioxidants in patients undergoing CPB (50-52).

*Phosphodiesterase Inhibitors*

The Medline search at the time of the study resulted in 17 citations for these phrases:

*“Cardiopulmonary Bypass” AND “inflammation” AND “phosphodiesterase inhibitors”*

Phosphodiesterase inhibitors are a group of drugs, which their role as anti-inflammatory agents has been under assessments for more than a decade (1). It has been demonstrated that the administration of phosphodiesterase inhibitors not only could decrease systemic vascular resistance (*i.e. *decreasing the afterload of the heart), but also, may prevent or alleviate the ensued myocardial dysfunction after CPB (53). Hence, the term “inodilator” has been coined for these agents. The mechanism of action in these agents is an increase in the level of the intracellular cyclic adenosine monophosphate (6). 

Pentoxifylline has been shown to “decrease the cellular need for energy” and “the cellular inflammatory reactions” through inhibition of 5›-nucleotidase (54). Morever, the same study showed that pentoxifylline can prevent “myocardial inflammation and I/R injury” in CPB. In another study performed in rats, the 4^th^ type of phosphodiesterase inhibitors could attenuate CPB related SIRS by regulating the pro-inflammatory mediators (55). However, this is not always the case and there are many controversial results in different studies regarding the role of phosphodiesterase inhibitors (56-59). One of the effects of milrinone is to improve the splanchnic circulation in patients undergoing CPB, hence, decreasing the level of systemic inflammatory mediators and preventing “gastric intramucosal acidosis” (59-60).

Finally, it should be said that milrinone is nowadays being used as a drug of choice in many cardiac surgeries using CPB; with a few claimed preventive and therapeutic effects. Suppression of the inflammation due to CPB might be one of them.

*The complement cascade*

The Medline search at the time of the study resulted in 172 citations for these phrases:

*“Cardiopulmonary Bypass” AND “inflammation” AND “complement”*

One of the most important players of inflammation, in all of the inflammatory processes (including during CPB) is the complement system. However, there are not a considerable number of therapeutic agents available to treat the effects of this system during CPB (61).

One of the main components of the complement system is a protein called C5. It has a major role in the complement cascade, since it can activate the remaining complement components. A recombinant antibody called pexilizumab can block C5; so, it might have a prominent role in suppressing the acute inflammatory response in CPB (62-64). However, the drug is not currently used as an “everyday medication”.

The components of the “complement cascade” and the “coagulation cascade” are highly inter-related. This relationship causes a number of effects, that is the final result of the sharing between the two cascades. The “direct enhancement of coagulation” due to the effects of the complement cascade in one hand, and the activation of the complement components due to the effects of coagulation enzymes on the other hand, are among the examples of this cross reactions; which would cause a number of inflammatory effects. These inflammatory effects are exactly the “especial position” for anti inflammatory drugs used during CPB (60-61).

Patients undergoing CPB are not the only example. There are a number of other disease states in which, the “complement-coagulation interaction” is the main cause for the creation of untoward lethal effects (60-63).

Although CPB related SIRS is the main cause of the inflammatory response (including the complement cascade), we should undoubtedly consider the role of surgery and anesthetic agents as the minor inevitable etiologies of SIRS. However, we have to consider that the role of anesthesia and surgery in production of inflammation is even more important in the pediatric population group (65).

*Miscellaneous agents*

Magnesium sulphate infusion is nowadays one of the commonly used anesthetic adjuvants (2-3). Analgesic effects, arrhythmia preventing and anti-arrhythmic properties, vasoconstriction and alleviation of increased pulmonary pressure are among its other applications. There are current studies that have demonstrated its use as an anti-inflammatory agent for patients undergoing cardiac surgery (3). The definite mechanism of action for magnesium sulphate is not yet clear; however, the role of magnesium as an antagonist of *N*-methyl-D-aspartate receptor (3) and also, a possible anti-apoptotic agent; through antagonizing intracellular calcium has been proposed (67).

Also, some of the anesthetic agents (including some of the volatile gases, a number of the opioids like morphine and, low-dose ketamine), some of the vasoactive agents (including dopexamine, sodium nitroprusside, angiotensin-converting enzyme inhibitors and levosimendane), erythropoietin, heparin and other glycosaminoglycans, statins, and H2 antagonists might suppress the inflammatory response related to CPB (5-6, 68-86).

*Non-pharmacologic strategies*

Heparin circuits, ultrafiltration and the issues related to ventilator-induced pulmonary inflammation are among the main non pharmacological issues that are not discussed here (87-111).

## Conclusion

The current evidence has introduced a wide range of therapeutic agents proposed for suppressing CPB related acute inflammatory status. However, none of these proposed therapeutic modalities are considered yet as the final solution for defying the inflammation in these patients. Hence, inflammation treatment in patients under CPB is still among the hot topics of research in this field. However, there is not enough evidence for most of these available agents as the choice of treatment. 
